# In-Line Detection with Microfluidic Bulk Acoustic Wave Resonator Gas Sensor for Gas Chromatography

**DOI:** 10.3390/s21206800

**Published:** 2021-10-13

**Authors:** Jizhou Hu, Hemi Qu, Wei Pang, Xuexin Duan

**Affiliations:** State Key Laboratory of Precision Measuring Technology and Instruments, College of Precision Instrument and Opto-Electronics Engineering, Tianjin University, Tianjin 300072, China; mrhjz1222@tju.edu.cn

**Keywords:** microfluidic channel, multi-dimensional gas chromatography, in-line detection, bulk acoustic wave resonator

## Abstract

A microfluidic film bulk acoustic wave resonator gas sensor (mFBAR) adapted specifically as an in-line detector in gas chromatography was described. This miniaturized vapor sensor was a non-destructive detector with very low dead volume (0.02 μL). It was prepared by enclosing the resonator in a microfluidic channel on a chip with dimensions of only 15 mm × 15 mm × 1 mm. The device with polymer coating showed satisfactory performance in the detection of organophosphorus compound, demonstrating a very low detection limit (a dozen parts per billion) with relatively short response time (about fifteen seconds) toward the simulant of chemical warfare agent, dimethyl methylphosphonate. The in-line detection of the mFBAR sensor with FID was constructed and employed to directly measure the concentration profile on the solid surface by the mFBAR with the controlled concentration profile in the mobile phase at the same time. The difference of peak-maximum position between mobile phase and solid phase could be a convenient indicator to measure mass transfer rate. With the response of the mFBAR and FID obtained in one injection, an injection mass-independent parameter can be calculated and used to identify the analyte of interest.

## 1. Introduction

Gas chromatography (GC) is the pillar technology for gas-phase analysis in a variety of applications including environmental science, clinic diagnosis, petroleum production, etc. [1,2,3,4]. To generate informative chromatogram, one or several detectors are utilized to measure the analytes eluting from the GC column. While the conventional detectors, such as thermal conductivity detectors (TCDs) [5] and flame ionization detectors (FIDs) [6], are generally installed at the terminal end, in-line detectors (or on-column detectors) could be configured in the capillary line, demonstrating a high flexibility in a serial combination with other detectors to provide multichannel detection with complementary information [7,8]. Additionally, an in-line detector, installed between separation columns, is capable of generating useful real-time information for the decision making of subsequent separations in the multidimensional GC (MDGC) [9,10,11]. However, besides microfluidic PID [12], in-line detection is rarely developed among conventional detectors, possibly because of the stringent requirements in the low dead volume and non-destructive characters.

Compared to the conventional GC, gas sensing technology offers a fast, on-site, and portable solution for the vapor analysis in the field. However, gas sensors implemented alone inevitably suffer from false alarms mostly due to their limitations in selectivity. Recent studies demonstrated that a combination of gas sensors with GC technology was promising to produce small-size and low-consumption instruments with good analytical accuracy. Therefore, many efforts have been dedicated to adapt gas sensors as GC detectors, including chemiresistors [13,14], chemicapacitor [15], surface plasmon resonance [16], Fabry–Pérot cavity sensor [17], optofluidic ring resonator [18], piezoelectric resonators [19], etc. In comparison with conventional GC detectors, the detectors based on micro gas sensors have the apparent advantage of a small footprint and are potential candidates for the in-line detection. For example, Fabry–Pérot cavity sensors could be directly used as in-line detectors [20,21]. Micro gas sensors, sealed in a microfluidic channel, could be conveniently employed for in-line detection with other types of detectors in a serial form [15,22].

Among gas sensors employed as the GC detectors, one approach is to measure the mass change induced by the adsorption/desorption of analyte in the efflux. This type of gravimetric sensors contains quartz crystal microbalance (QCM) [23], a surface acoustic wave (SAW) device [24,25,26,27], and cantilevers [28]. Being similar with QCM, the film bulk acoustic wave resonator (FBAR) is a microelectromechanical system (MEMS)-based bulk acoustic wave micro device. According to the Sauerbrey equation, a FBAR sensor, resonating at the gigahertz range, offers higher sensitivity than QCM, which has the resonating frequency at several megahertz [29,30,31,32,33]. We are quite interested in applying FBAR gas sensors as GC detectors and have firstly reported the facile hyphenation of the FBARs with GC [34,35]. In this study, we aimed to develop a microfluidic FBAR (mFBAR) gas sensor for in-line detection in GC. This mFBAR-based GC detector has a nearly zero dead volume and a concise fluidic design. A direct connection of the mFBAR with FID produced a tandem hybrid detection system, which generated FID chromatogram-containing information of the mobile gas phase and the mFBAR chromatogram, reflecting the adsorption/desorption on the solid phase. Comparison study between FID and the mFBAR chromatogram reveals not only the inherent character of the mFBAR, but also the adsorption/desorption behavior of vapors on the surface. In the end, to demonstrate the utility of an mFBAR gas sensor in MDGC, a preliminary GC–GC system, with the mFBAR installed between separation columns, was configured and tested in a 11-component mixture.

## 2. Materials and Methods

### 2.1. Device Fabrication

The 2.44 GHz FBARs were fabricated in the clean room by MEMS technology. The fabrication process has been reported in our previous work [36] and is briefly described as in the following steps (see Figure S1). Step 1: an air cavity was etched on the silicon substrate by deep-reactive ion etching (DRIE). Step 2: the sacrifice material (phospho-silicate glass, PSG) was filled into the air cavity by chemical vapor deposition (CVD). Step 3: a thin layer of molybdenum (Mo) was deposited on the silicon substrate as the bottom electrode by evaporation. Step 4: piezoelectric aluminum nitride (AlN) film (390 nm) was deposited by sputtering. Step 5: a layer of Mo was fabricated to form the top electrode by a lift-off process. Step 6: via holes were etched to connect to the bottom electrode and the diluted HF solution was introduced to remove the sacrificial material. FBARs were fabricated on the wafer scale and the size of a single device was approximately 1 mm × 1 mm. To enhance vapor analyte sorption, a solution of polymer (Polyethyleneimine, PEI) could be sprayed onto the top electrode to form a thin sorptive layer by ink-jet printing (Jetlab 4, MicroFab, Plano, TX, USA).

**Figure 1:**
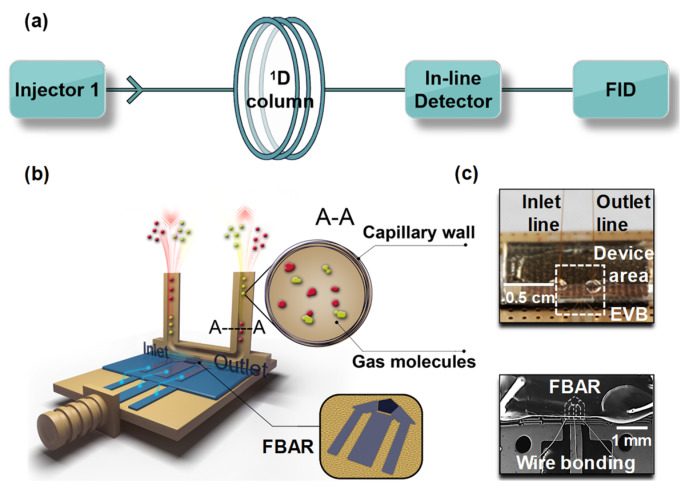
Simplified instrument configuration diagram showing the facilely hyphenated GC (**a**). (**b**) Cartoon showing the prototype microfluidic FBAR detector in a gas chromatography system. (**c**) The digital photo and scanning electron microscope picture showing the microfluidic FBAR detector.

The microfluidic channel was fabricated on a transparent glass through laser-induced thermal etching (DPU-10, Shenzhen Laser Technology, Shenzhen, China). This channel was a cuboid groove along one side with two through holes to the other side. The dimensions of the groove were 4 mm in length, 250 μm in width, and 20 μm in depth. The shallow groove in the glass chip was oppositely flip-attached to the FBARs chip and the joint was sealed with epoxy glue, thus encapsulating the FBAR sensor (Figure 1b and Figure S1). The gas inlet and outlet were formed by inserting capillary tubes in the two through holes and the joints were also sealed with epoxy glue. This microfluidic channel capped FBARs chip with capillary connection was wire-bonded on an evaluation board (EVB) to mFBAR gas sensors and used as the detector in subsequent gas chromatography experiments (Figure 1c).

### 2.2. Gas Sensing Experiments

A dynamic gas distribution instrument (MF-3D, National Institute of Metrology, China) was used to produce ethanol vapors (12, 24, 48, 72 ppm), acetone (8.65, 17.3, 34.6, 51.9 ppm), heptane (4.6, 9.2, 18.4, 27.6 ppm), toluene (4.6, 9.2, 18.4, 27.6 ppm) and DMMP (dimethyl methylphosphonate, the simulant of chemical warfare agent sarin) vapors (0.332, 0.664, 1.328, 1.992 ppm). The vapors are then guided into the testing chamber, where we place the bare and PEI-coated FBAR sensors. The response of FBARs was measured by a vector network analyzer and recorded by a MATLAB program in real time. The nitrogen gas is sequentially purged into the chamber until the equilibrium response.

### 2.3. GC–mFBAR–FID System

A commercial bench-top GC apparatus (7890C, Agilent technology, Santa Clara, CA, USA) was used to characterize microfluidic FBAR detectors. As shown in Figure S2, The GC experiments were performed with an automatic split/splitless injector, a separation column, an in-line detector, and a flame ionization detector (FID). Helium was used as the carrier gas with flow rates between 0.5 and 10 mL min^−1^. The injector was operated at 250 °C with an injection volume of 1.0 μL at a split ratio of ~10:1, unless otherwise stated. The oven temperature was set as T_initial_ = 50 °C, ramp = 10 °C min^−1^, T_final_ = 230 °C. HP-5ms (30 m × 0.32 mm ID, 0.5 μm, Agilent technology) was used as a separation column in the oven. The microfluidic FBAR detector was installed immediately after the column as an in-line detector outside the oven. The inlet and outlet of microfluidic FBAR detector were connected to the separation column and FID through transfer lines with pressfit unions (5190–6979, Agilent technology, Santa Clara, CA, USA). The transfer line was a deactivated fused silica column (0.3 m × 0.32 mm). The resonant frequency of the FBAR detector was continuously monitored by a vector network analyzer (E5061B, Keysight, Santa Rosa, CA, USA) and recorded through a custom-made MATLAB program on a personal laptop. The data acquisition rate of the FBAR resonance frequency is 20 points per min. or 60 points per min., to ensure 10 points per peak at least in the chromatogram. The FID was operated at a constant temperature of 250 °C with airflow of 350 mL min^−1^ and hydrogen flow of 35 mL min^−1^.

## 3. Results and Discussion

### 3.1. Gas Sensing Performance

The response of a FBAR detector is governed by the Sauerbrey equation, which defines that the frequency change is directly proportional to the added mass on the top electrode, as follows [37,38]:

(1) $$\Delta f=-\frac{2f_{0}^{2}}{A\sqrt{\rho_{q}\mu_{q}}}\Delta m,$$

(2) Δf=−B·Δm,

where Δf and $f_{0}$ are the frequency shift and the resonant frequency of FBAR, respectively; $\mu_{q}$ and $\rho_{q}$ are the elastic modulus and the density of piezoelectric material, respectively; A is the area of the plate; Δm is the added mass; B is a constant for a specific FBAR device. The Sauerbrey equation is valid as long as the added mass is relatively small in comparison with the mass of piezoelectric layer and rigid. The adsorption quantity on the solid surface is expressed by the following equation [39]:

(3) $$K_{T}=\frac{C_{s}}{C_{g}}=\frac{\Delta n_{s}/A}{C_{g}}=\frac{\Delta m_{s}}{M_{w}\cdot C_{g}\cdot A},$$

where $K_{T}$ is the equilibrium constant at the temperature of *T*; $C_{s}$ and $C_{g}$ are the analyte concentrations on the solid surface and in the gas phase, respectively; A is the exposed area of the top electrode of FBAR in the microfluidics.

In order to evaluate the performance of FBAR sensors in the vapor analysis, we investigated the sensitivity, linearity, limit of detection (LOD) and stability. The polymer PEI was chosen as the coating material in the sensor since this polymer is commercially available and capable of providing hydrogen bonding by large numbers of imine groups. In addition, our preliminary results have shown that it could be a potential candidate as sensitive materials for the detection of chemical warfare agents [40]. The response here is defined as the maximum response of the analyte.

**Figure 2:**
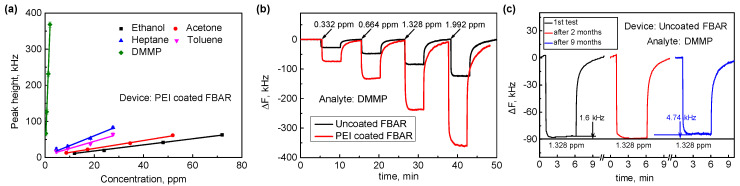
(**a**) The linear relationship between the response of PEI–coated FBAR sensor and the concentration of various vapors. (**b**) Reversible response of uncoated FBAR and PEI–coated FBAR sensor to DMMP with concentrations at 0.332, 0.664, 1.328 and 1.992 ppm. (**c**) Stability of uncoated FBAR sensor at ambient condition in 9 months.

In Figure 2a, we plot the response of the PEI-coated mFBAR with vapor concentration for five analytes. The vapor concentration was varied from part per billion (ppb) to part per million (ppm). The sensitivity of the PEI-coated mFBAR toward ethanol, acetone, heptane, toluene and DMMP were 0.84038, 1.09487, 2.7376, 1.98344 and 178.7276 kHz ppm^−1^. Figure 2b compared the real-time response of uncoated FBAR and PEI-coated FBAR toward DMMP of varied concentration. The sensor with PEI coating demonstrates the enhanced DMMP sensitivity of nearly three times as high as the uncoated mFBAR. However, the sensitivity toward the other vapors did not show significant change after coating. As can be seen in Table 1, the calculated sensitivity of the PEI-coated mFBAR is very close to the calculated sensitivity of uncoated FBAR toward ethanol, acetone, heptane and toluene. This trend indicates a favorable interaction between PEI and DMMP.

Then, we inspected the linearity. In Figure 2a and Figure S3, the responses of the two FBAR sensors with the concentration show excellent linearity with an R^2^ of 0.97592–0.99774 and negligible interception (less than 3 kHz) in the linear regression analysis for all vapors. This linearity indicates a constant value of $K_{T}$ at the value of less than 5% of saturation vapor pressure (*p*_0_). It should be noted that the value of $K_{T}$ could vary gradually for the higher concentration according to the Langmuir model and the Freundlich model [41].

The LODs for the tested vapors are directly calculated as minimum exposure concentration required to generate the response at the signal-to-noise ratio (S/N) of 3. The noise is defined by the two parallel lines drawn between the peak-to-peak maxima and minima from the baseline over a period of time, which are calculated to 0.8 kHz at the operation temperature. The results are calculated and summarized in Table 1. The polymer-coated mFBAR in the exposure of DMMP shows a theoretical LOD as low as 1 ppb and a verified value of 13.4 ppb as GC detector. The injected mass in GC could be converted to average gas-phase dimensionless concentration with the following equation [22]:

(4) $$c=\frac{c_{1}V_{1}V_{m}S_{R}}{M_{W}F\Delta t},$$

where $c_{1}$ is the mass density of DMMP in the liquid sample, $V_{1}$ is the liquid volume of sample injected into the column, $V_{m}$ (=24.0 L/mol) is the molar volume of an ideal gas at the outlet temperature (20 °C), $S_{R}$ is the injection split ratio, $M_{W}$ is the molecular weight of the analyte, Δt is the peak width in time, and F is the column flow rate. The detectable minimum injection mass of DMMP for the polymer-coated mFBAR is 0.89 ng, which corresponds to an average gas-phase concentration of 13.4 ppb according to Equation (4).

To assess the stability of the FBAR gas sensor, the same sensor was repeatedly tested in the DMMP vapor within nine months. Figure 2c shows the stability test of uncoated FBAR gas sensors. In comparison with the initial response of sensor in DMMP vapor, the sensor produces a slightly lower sensing response (<4%) to the vapors of identical concentration, indicating good stability of the sensor. This result demonstrated that the device is stable at ambient condition and the stability in sensor response is mainly dependent on the stability of sorption materials. Figure S4 shows the stability test of PEI-coated FBAR at ambient condition. We did not observe a significant change in response, indicating a good stability of PEI-polymer coating in nine months.

### 3.2. Flow Profile in mFBAR Sensor

**Figure 3:**
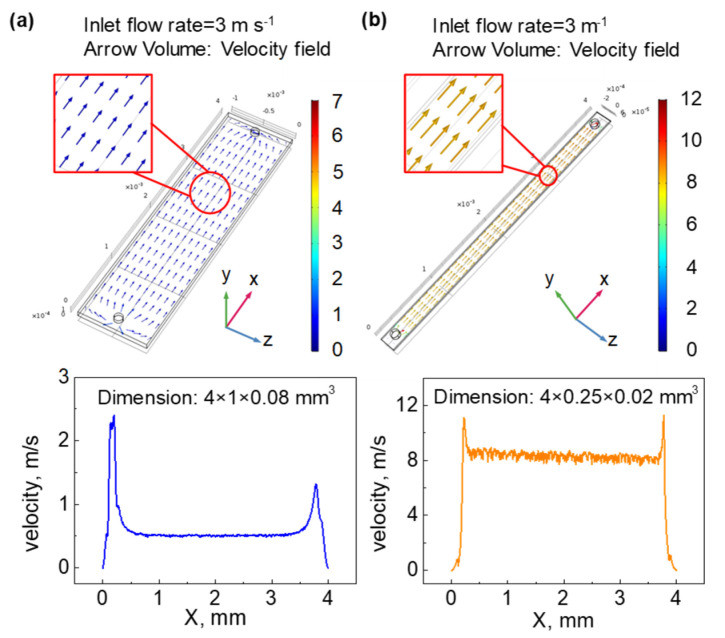
COMSOL simulation of analyte (ethanol) flow rate magnitude for different sizes of gas chambers. (**a**) 4 × 1 × 0.08 mm^3^. (**b**) 4 × 0.25 × 0.02 mm^3^. The data graph below shows the velocity change of ethanol vapor in the x-axis direction within the microchannel. The diameter of the inlet and outlet is 0.12 mm. In the beginning, the chamber is full of ethanol homogeneously. Helium, as purging gas, is flowed in at t = 0 with a flow rate of 3 m s^−1^ to purge the gas chamber. The dead volume, defined as the space inside the chamber, is calculated to be 0.32 μL and 0.02 μL, respectively, for (**a**,**b**).

To understand the influence of the in-line detector on the capillary flow profile, we carried out the COMSOL simulation toward the gas flow passing through the mFBAR detector. Figure 3 shows the plots of the flow rate against the length of a microfluidic channel. As expected, the microfluidic channel with the cross section in a similar size as the inlet hole offers a more even flow profile (Figure 3b). Under the device of 4 × 0.25 × 0.02 mm^3^ dimension, the velocity inside the microchannel is about 8 m s^−1^ when 3 m s^−1^ of helium gas from the inlet is applied. Next, we studied the flow disturbance after the introduction of the microfluidic FBAR by GC experiments. By using FID as a terminal detector to record chromatograms, we compared the signals before installing the microfluidic FBAR detector with that after installing the microfluidic FBAR detector. As shown in Figure S5, those two chromatograms are nearly identical except for a minor shift in retention time, demonstrating that the effect of introducing a microfluidic FBAR detector is equivalent to that of introducing a transfer line. This quasi “transfer line” is too short to affect the separation efficiency in our system.

### 3.3. In-Line Detection of mFBAR Sensor with FID

With in-line detection of the mFBAR sensor with FID, both the FID chromatogram and the mFBAR chromatogram can be obtained. While the FID chromatogram reveals the information in the gas phase, the mFBAR chromatogram offers the information on the solid surface. For a FID device, the magnitude of signal is directly proportional to the concentration of analyte in the mobile gas phase. In comparison with FID, the FBAR detector operates on measuring the change in the weight of adsorbates on the solid surface rather than the quantity of analyte in the mobile phase. This kind of complementarity is beneficial in understanding the thermodynamic and dynamic for the interaction between gas analytes and the solid surface since the adsorption/desorption process involves the mass transfer between the gas phase and solid phase.

**Figure 4:**
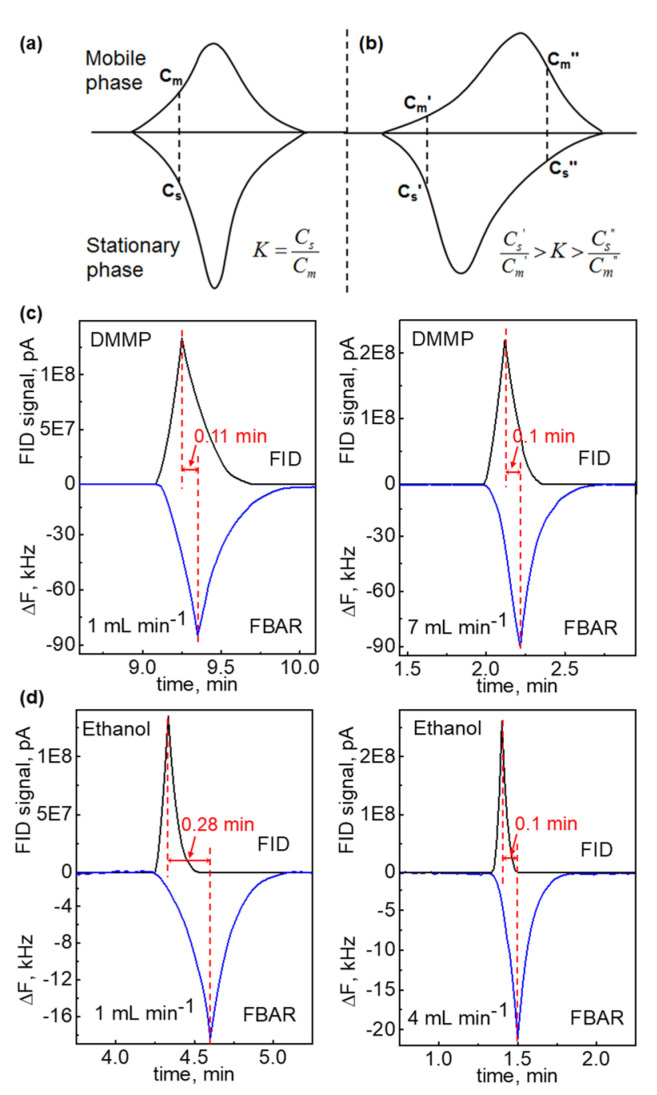
The measurement of the concentration profile on the solid surface by the PEI–coated mFBAR with the controlled concentration profile in the mobile phase by FID at the same time. (**a**,**b**) Simulated analyte concentration profile in the mobile gas phase (top) and in the stationary phase (bottom) for the fast mass transfer (**a**) and slow mass transfer (**b**). *K* is the equilibrium constant; $C_{s}$, $C_{s}^{\prime }$, $C_{s}^{\prime \prime }$, $C_{m}$, $C_{m}^{\prime }$ and $C_{m}^{\prime \prime }$ are the analyte concentration on the solid surface and in the mobile phase, respectively. (**c**,**d**) The comparison of chromatogram from FID (top) with that from the PEI–coated mFBAR (bottom) at the different flow rates for analyte DMMP (**c**) and ethanol (**d**).

In-line detection of the mFBAR sensor with FID could help to reveal the mass transfer process in the dynamics of chromatography. Traditionally, chromatographers analyze the dynamics without the information of concentration profile in the stationary phase. In-line detection of the mFBAR sensor with FID offers the opportunity of the direct measurement of the concentration profile on the solid surface by the mFBAR with the controlled concentration profile in the mobile phase by FID at the same time. In the GC–mFBAR–FID system, the PEI-coated mFBAR detector is firstly exposed to the analytes in the GC effluent, describing the concentration profile on the solid surface by measuring the mass changing induced by the adsorption/desorption process. Later, the terminal FID directly ionizes the vapor analytes and measures the concentration profile in the mobile phase. The pattern in the vapor concentration can be easily varied by changing the flow rate. Here, we compared the chromatogram of FID with the PEI-coated mFBAR. It should be noted that, through changing the coating material, the same procedure could be applied to analyze interaction of analytes with any other popular stationary phase materials such as OV-1, SE-30, etc. Figure 4a,b shows the simulated concentration profile of the two analytes in the mobile gas phase (top) and in the stationary phase (bottom) for the fast mass transfer and slow mass transfer. In the fast mass transfer, the peak maximums in the mobile phase and solid phase are located at the same position because of reaching equilibrium quickly. In the slow mass transfer, the peak maximums are far away from each other, where the equilibrium is not achieved. In Figure 4c, we show that the mass transfer of DMMP between the mobile gas phase and the solid surface reaches equilibrium quickly, thus giving the peak maximum at the closed position in the concentration profile when the flow rate is 1 mL min^−1^. The flow rate appears to have an insignificant effect on the mass transfer between DMMP and PEI polymer since there is no significant difference of concentration profile from 1 mL min^−1^ to 7 mL min^−1^. As can be seen in Figure 4c, the difference in peak position remains the same when the flow rate is 7 mL min^−1^ for DMMP. In comparison, shown in Figure 4d, the adsorption/desorption of ethanol between the mobile gas phase and the solid surface deviate significantly from the equilibrium due to a relatively slow mass transfer rate when the flow rate is 1 mL min^−1^, giving the different peak maximum positions far away from each other. The flow rate appears to have a significant effect on the mass transfer between ethanol and PEI polymer. The difference in peak position for ethanol reduces from 0.283 (1 mL min^−1^) to 0.1 (4 mL min^−1^). The mass transfer of ethanol would increase to the saturation when the flow rate increases to 4 mL min^−1^. After 4 mL min^−1^, the magnitude of variation for ethanol is the same as that for the DMMP, indicating a similar mass transfer rate. The amplitude of difference in peak maximum positions could be a convenient indicator of mass transfer rate. We summarized the value of variation for the two vapors at the different flow rates in Table S1.

In-line detection of the mFBAR sensor with FID could be used to generate analyte-specific parameters, which could be further used for analyte identification. Firstly, we calculated the amount of analytes in the mobile gas phase by using FID peak area with the equation:

(5) $$P_{A}=R\cdot n_{A},$$

where $P_{A}$ is the peak area of analyte, R is response factor, which is a constant for a specified GC, $n_{A}$ is the amount of analytes. Then, we compared the response of FID and the response of the mFBAR to give analyte-specific parameter S as the following:

(6) $$S=\frac{\Delta f}{P_{A}}=\frac{-B\cdot M_{W}\cdot \Delta n_{s}}{R\cdot n_{A}},$$

where Δf is the peak height of analyte. In this equation, the obtained *S* is related to the molecular weight of the analyte $M_{W}$ and response factor R. In addition, it is observed that Δf and $P_{A}$ are linearly proportional to the injected mass in the range of 3.14–94.2 ng for DMMP and 19.7–591 ng for ethanol, and the curves pass through the origin point (see Figure S6). Therefore, the obtained *S* is also injection mass-independent in the linear ranges. It could be calculated in one injection thanks to the in-line detection of the mFBAR with FID. In Table 2, we summarized the obtained *S* values. Among them, DMMP gave the largest value and heptane showed the minimum. The dependence of this parameter (*S*) on the flow rate is also investigated. For ethanol and DMMP, the variation is less than 5% when the flow rate is in the range of 1–7 mL min^−1^ (See Table S2). Therefore, we could use this parameter to identify the analyte of interest when *S* is known for this specific analyte.

**Figure 5:**
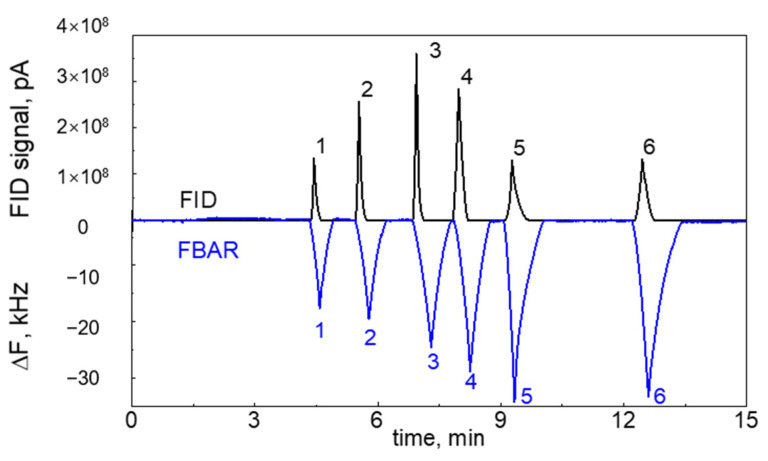
Chromatographic separation and mFBAR sensor as well as FID detection of six mixed chemical compounds. Simultaneous chromatogram traces from a mFBAR sensor (blue, bottom) and a downstream FID (black, top). 1: Ethanol; 2: Acetone; 3: Toluene; 4: Heptane; 5: DMMP; 6: MS. The proportion of the six analytes was 1 μL: 1 μL: 1 μL: 1 μL: 0.1 μL: 0.1 μL, split ratio = 10:1. The oven temperature was set as T_initial_ = 50 °C, ramp = 15 °C min^−1^, T_final_ = 230 °C. Constant helium gas flow = 1 mL min^−1^.

**Figure 6:**
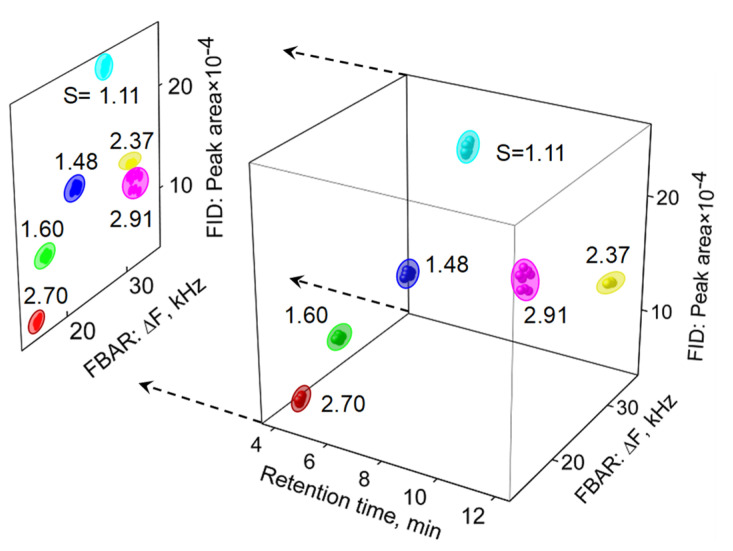
Three-dimensional recognition and two-dimensional projection plots of the 6 analytes using the mFBAR peak sensor response, FID response, and the GC retention time as the three axes. Each cluster corresponds to nine independent measurements. The *S* value was labeled alongside the analyte cluster.

Figure 5 displays two simultaneous chromatograms which were obtained from the mFBAR sensor as well as the downstream FID. Combined with the diverse response pattern of the mFBAR and FID with GC separation, the recognition ability for various analytes was significantly improved by using the analyte-specific parameter *S*. Nine repeated injections of six chemicals were introduced into the in-line GC system. The resulting data, including responses from both detectors and retention time passing through the column, was recorded and presented in a three-dimensional space, subsequently projected onto the left plane generated by employing only the responses from FID and FBAR. As shown in Figure 6, the six analytes occupied different and isolated volumes in three-dimensional and even two-dimensional recognition space, indicating a facile identification by use of the analyte-specific parameter *S*.

### 3.4. Application in GC–GC Separation

**Figure 7:**
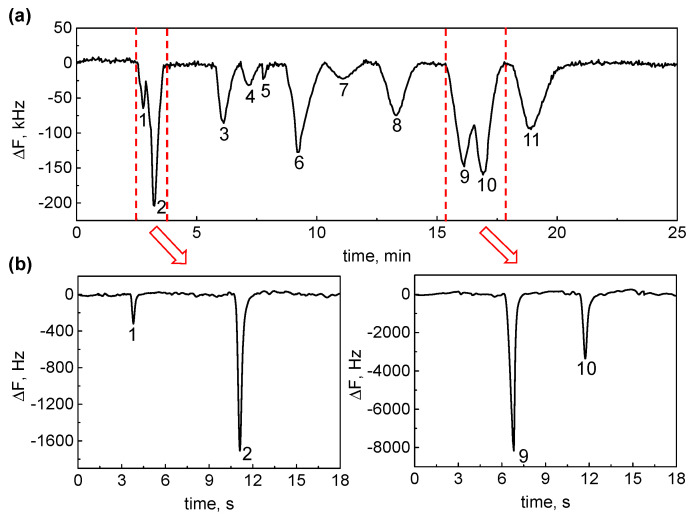
(**a**) Real–time response of eleven gases detected by 1D GC. 1: Ethanol; 2: DCH; 3: Acetone; 4: TCH; 5; Hexane; 6: Benzene; 7: Toluene; 8: Heptane; 9: DMMP; 10: DIMP; 11: MS. The proportion of the eleven analytes was 0.3 μL: 0.45 μL: 0.3 μL: 0.1 μL: 0.1 μL: 0.3 μL: 0.1 μL: 0.1 μL: 0.05 μL: 0.05 μL: 0.05 μL. The oven temperature was set as T = 230 °C. Constant helium gas flow = 0.5 mL min^−1^. (**b**) Heart–cut 2D detection of two pairs of unseparated analytes.

To demonstrate the usefulness of the mFBAR detector in the MDGC, we constructed a preliminary GC–GC system. The system set-up was described in the supporting information and shown in Figure S7. Figure 7a shows the 1D separation chromatogram of 11 compounds by using the mFBAR detector as an in-line detector. It should be noted that this chromatogram contains separation information of the heart-cut portions in the 1D separation column, while those information elements are generally missed in conventional heart-cut 2D GC. With the real-time information provided by the in-line detector, the heart-cutting times can be determined and then used in the time-oriented sequential control program to automatically cut the desired 1D effluent portions into a 2D separation column. Figure 7b shows the 2D separation chromatogram of two heart-cut windows from the 1D separation column. As shown in Figure 7b, two pairs of analytes, difficult to be separated in the 1D column, are separated thoroughly by a fast 2D separation column operated in the heart-cut model after in-line detection. Additionally, we observed that the ratio of the intensity changed after 2D separation for the two pairs (1 and 2, 9 and 10). This change is caused by injector of the 2D chromatography, where the concentrator shows the different trapping efficiency toward the analytes (1 and 2, 9 and 10) and some vapors penetrated the concentrator.

## 4. Conclusions

In this work, we developed a novel microfluidic FBAR for the in-line detection in GC. In this detector, FBAR is enclosed in the microfluidic channel as the detection element and works on an adsorption/desorption mechanism. Comparative experiments and simulation demonstrated that installing an additional microfluidic FBAR in the capillary line did not cause any significant flow disturbance and thus deteriorate separation. In the GC–mFBAR–FID system, we demonstrated that in-line detection of the mFBAR sensor with FID could directly measure the concentration profile on the solid surface by the mFBAR with the controlled concentration profile in the mobile phase by FID at the same time. The calculated parameter based on the response of the mFBAR and FID was observed to be nearly constant for a specific analyte regardless of concentrations and flow rates in the linear response range. This work shows the potential for chromatographers to analyze the dynamics with the information of the concentration profile in the mobile gas phase by FID and on the solid surface by the mFBAR as well as to utilize the mFBAR with the time-oriented sequential control program to automatically cut the desired 1D effluent portions into a 2D separation column.

## Figures and Tables

**Table 1:** Sensitivity and LOD Parameters for FBAR detectors in the detection of five VOCs.

Device	PEI—Coated FBAR		Uncoated FBAR	
Parameters	Sensitivity, kHz ppm^−1^	LOD, ppm	Sensitivity, kHz ppm^−1^	LOD, ppm
Ethanol	0.840	2.856	0.551	4.356
Acetone	1.095	2.192	0.870	2.759
Heptane	2.738	0.877	2.500	0.960
Toluene	1.983	1.210	1.791	1.340
DMMP	178.728	0.013	60.313	0.040

**Table 2:** *S* values for the six vapors.

Vapors	Ethanol	Acetone	Toluene	Heptane	DMMP	MS
S (10^7^)	2.70	1.60	1.48	1.11	2.91	2.37
Hz (A·s)^−1^						

## Data Availability

Not applicable.

## Supplementary Materials

The following are available online at https://www.mdpi.com/article/10.3390/s21206800/s1, Figure S1: Fabrication process of mFBAR, Figure S2: GC–mFBAR–FID system set-up, Figure S3: Linearity of uncoated FBAR sensor for various vapors, Figure S4: Stability test of PEI-coated FBAR, Figure S5: Flow disturbance, Table S1: Variation value of peak maximum positions for the two vapors at the different flow rates, Figure S6: Response of mFBAR and FID for ethanol and DMMP, Table S2: The dependence of S value on the flow rates for ethanol and DMMP. Figure S7. Cartoon showing the prototype heart-cut two-dimensional GC system.
