# Supplementary material for: In-Line Detection with Microfluidic Bulk Acoustic Wave Resonator Gas Sensor for Gas Chromatography
Source: Sensors (Basel). 2021 Oct 13;21(20):6800. doi: 10.3390/s21206800 (PMC8540273; doi:10.3390/s21206800)
Supplement: Supplementary file 1 [file sensors-21-06800-s001.zip › sensors-1411709-supplementary.pdf]

sensors

Supporting Information

In-line Detection with Microfluidic Bulk Acoustic Wave Resonator Gas Sensor for Gas Chromatography

Jizhou Hu, Hemi Qu*, Wei Pang* and Xuexin Duan*

State Key Laboratory of Precision Measuring Technology and Instruments, College of Precision Instrument and Opto-electronics Engineering, Tianjin University, Tianjin 300072, China; mrhjz1222@tju.edu.cn
* Correspondence: hemi.qu@tju.edu.cn (H.Q.); weipang@tju.edu.cn (W.P.); xduan@tju.edu.cn (X.D.); Tel.: +86-22-2740-1002 (H.Q. & X.D.)

Citation: Hu, J.; Qu, H.; Pang, W.; Duan, X. In-line Detection with Microfluidic Bulk Acoustic Wave Resonator Gas Sensor for Gas Chromatography. *Sensors* **2021**, *21*, 6800. https://doi.org/10.3390/s21206800

Academic Editor: Tomasz Marcin Dymerski

Received: 22 September 2021
Accepted: 11 October 2021
Published: 13 October 2021

Publisher's Note: MDPI stays neutral with regard to jurisdictional claims in published maps and institutional affiliations.

Copyright: © 2021 by the authors. Submitted for possible open access publication under the terms and conditions of the Creative Commons Attribution (CC BY) license (http://creativecommons.org/licenses/by/4.0/).

1. Chemicals

VOCs (benzene, toluene, n-hexane, n-heptane, acetone, ethanol, dichloromethane (DCH), and trichloromethane (TCH)) and CWA simulants (dimethyl methyl phosphonate (DMMP), diisopropyl methylphosphonate (DIMP) and methyl salicylate (MS)) were obtained from Tianjin Yuanli Chemical Corporation (Tianjin, China). The purity of above-mentioned chemicals all reached HPLC.

2. Fabrication Process of mFBAR

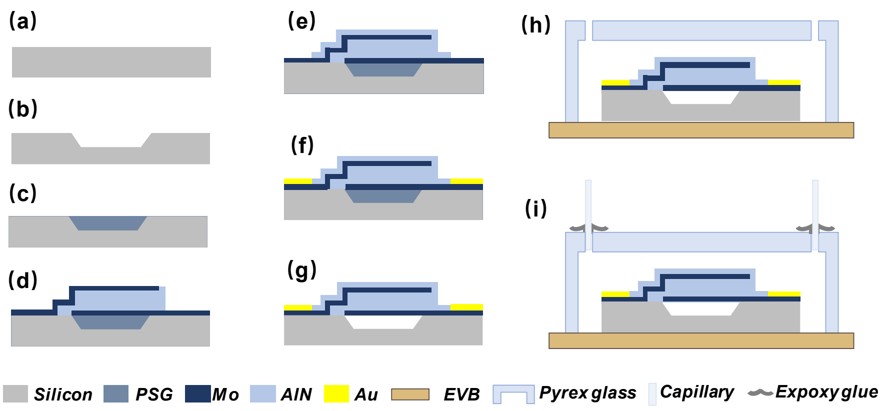

Figure S1. MEMS technology based fabrication process of microfluidic FBAR device. (**a**) The silicon wafer was rinsed with deionized water for the next steps. (**b**) An air cavity was etched on the silicon substrate by deep–reactive ion etching (DRIE). (**c**) The sacrifice material (phospho–silicate glass, PSG) was filled into the air cavity by chemical vapor deposition (CVD). (**d**) A thin layer of molybdenum (Mo) was deposited on the silicon substrate as the bottom electrode by evaporation. Then the piezoelectric aluminum nitride (AlN) film (390 nm) was deposited by sputtering and a layer of Mo was fabricated to form the top electrode by a lift-off process. (**e**) The passivation layer was deposited above. (**f**) The Au pads were evaporated and patterned to serve as electrical connections. (**g**) Via holes were etched to connect to the bottom electrode and the diluted HF solution was introduced to remove the sacrificial material. (**h**) A shallow groove etched on a transparent glass with two through holes was oppositely flip–attached to the FBAR chip and the joint was sealed with expoxy glue. (**i**) The gas inlet and outlet were formed by inserting capillary tubes in the two through holes and the joints were also sealed with expoxy glue.

3. GC-mFBAR-FID System Set-up.

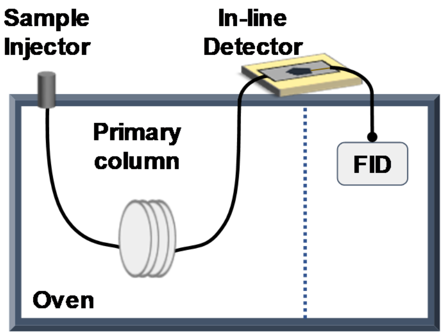

Figure S2. Cartoon showing the prototype GC-mFBAR-FID system.

4. Linearity of Uncoated FBAR Sensor for Various Vapors

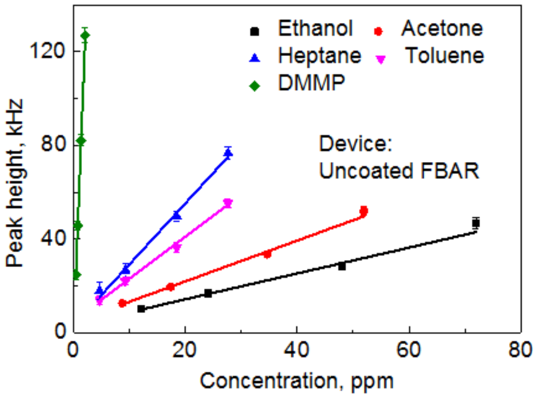

Figure S3. The linear relationship between the response of uncoated FBAR sensor and the concentration of various vapors.

5. Stability Test of PEI-coated FBAR

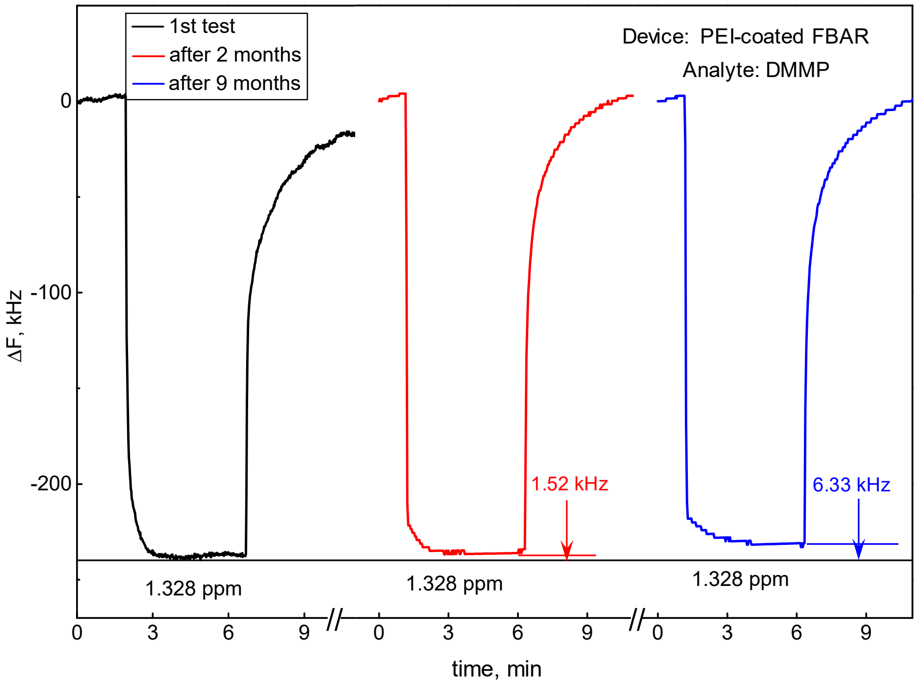

Figure S4. Repeatedly test in the DMMP vapor for PEI coated FBAR at the average concentration of 1.328 ppm within nine months.

6. Flow Disturbance

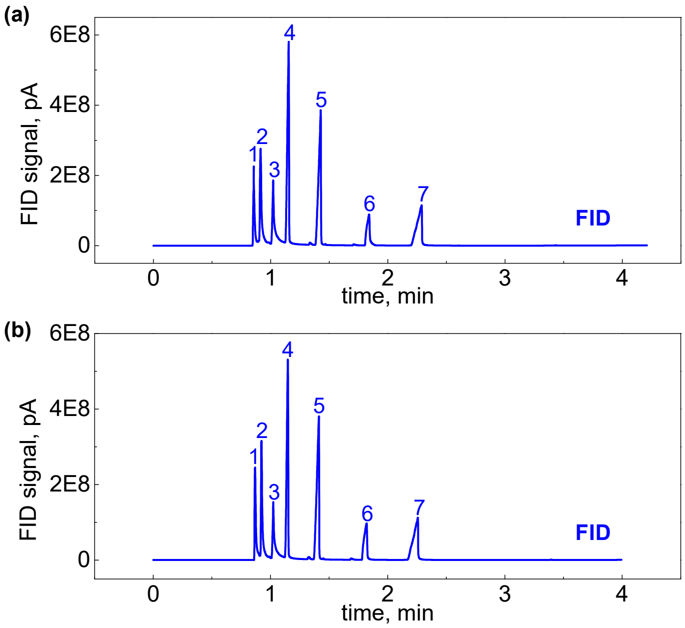

Figure S5. Contrast curve of gas chromatographic separation and detection (**a**) before and (**b**) after installing the microfluidic FBAR detector in front of FID. 1: Ethanol; 2: Acetone; 3: Hexane; 4: Toluene; 5: Heptane; 6: DMMP; 7: MS. The proportion of the seven analytes was 1 μl: 1 μl: 1 μl: 1 μl: 1 μl: 0.1 μl: 0.1 μl.

Table S1. Variation value of peak maximum positions for the two vapors at the different flow rates.

Flow rates/mL min^{-1}	1	2	3	4	5	6	7
DMMP, min	0.11	0.11	0.11	0.1	0.1	0.1	0.1
Ethanol, min	0.28	0.20	0.13	0.1	0.1	0.1	0.1

7. Response of mFBAR and FID for Ethanol and DMMP

We define that A_1 is the slope of Δf from the mFBAR sensor and that of A_2 is for the FID signal. According to the experiment results, it is observed that the value of S is equal to A_1/A_2 in the linear ranges shown in Figure S6. The regressive equation for mFBAR toward DMMP in Figure S6(a) was $y = A_1x + 1.843$, where A_1 was 0.992. The regressive equation for FID toward DMMP in Figure S6(a) was $y = A_2x + 0.472$, where A_2 was 0.351. The regressive equation for mFBAR toward ethanol in Figure S6b was $y = A_1x + 1.248$, where A_1 was 0.075. The regressive equation for FID toward ethanol in Figure S6(b) was $y = A_2x + 0.108$, where A_2 was 0.028.

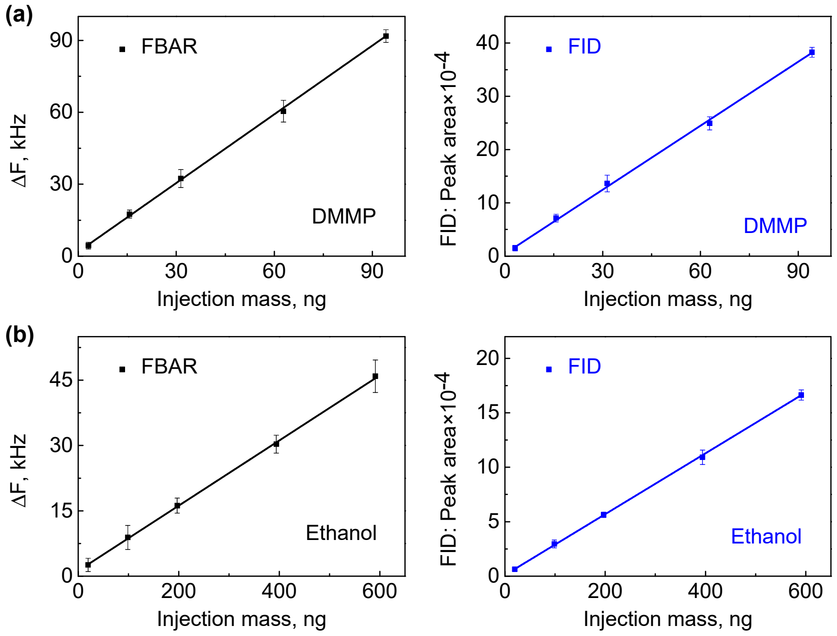

Figure S6. The linear relationship between the response of mFBAR sensor as well as FID and the injection mass of (**a**) DMMP and (**b**) ethanol.

Table S2. The dependence of S value on the flow rates for the ethanol and DMMP.

Vapors \ Flow rates, mL min^{-1}	1	2	3	4	5	6	7
DMMP (10^7)	2.91	2.95	2.94	2.99	2.98	2.92	3.01
Ethanol (10^7)	2.70	2.76	2.68	2.80	2.78	2.79	2.73

8. Preliminary Two-Dimensional GC System

The preliminary two-dimensional GC experiments were performed on the Agilent bench-top 7890C GC coupled with a fast portable GC (zNose 4300, Electronic Sensor Technology, USA). In this set-up, the 1D separation system used the same configuration described in the GC-mFBAR-FID system paragraph including an injector, a 1D separation column, and an in-line mFBAR detector, except that FID detector was removed in the terminal. The 2D separation used a DB-5 column (1 m × 0.32 mm ID, 0.5 μm, Agilent technology) which was integrated in the zNose 4300. The interface between the in-line mFBAR detector and 2D separation column mainly consisted of a 3-port valve, a preconcentration collector, and a 6-port valve. The transfer line also used deactivated fused silica column (0.3 m × 0.32 mm) with pressfit unions to connect the in-line mFBAR detector, 3-port valve, and preconcentrator collector. The injector in 1D separation system was maintained at 250 °C with an injection volume of 1.0 μL at a split ratio of ~10:1. The oven temperature in 1D separation system was set at $T_{initial}$ = 50 °C, ramp = 10 °C min^{-1}, T_{final} = 230 °C. The column temperature in 2D separation system was set at $T_{initial}$ = 50 °C, ramp = 10 °C s^{-1}, T_{final} = 200 °C. Helium flow in the 1D separation system was maintained at the rate of 2 mL min^{-1}. Helium flow in the 2D separation system was provided through a 6-port valve at the rate of 3 mL min^{-1}. The 3-port valve was normally maintained at a position of preventing flow from the 1D separation system to the 2D separation system. The heart-cutting fraction was obtained by defining the time of switching on/off based on the information from the in-line detector. This fraction was firstly trapped at 20 °C on the preconcentration collector and then released at 200 °C. The thermal desorption fraction was sampled into the 2D column through the 6-port valve for further separation. The effluents from the 2D column

were continuously monitored by a surface acoustic wave detector which was integrated in the zNose 4300.

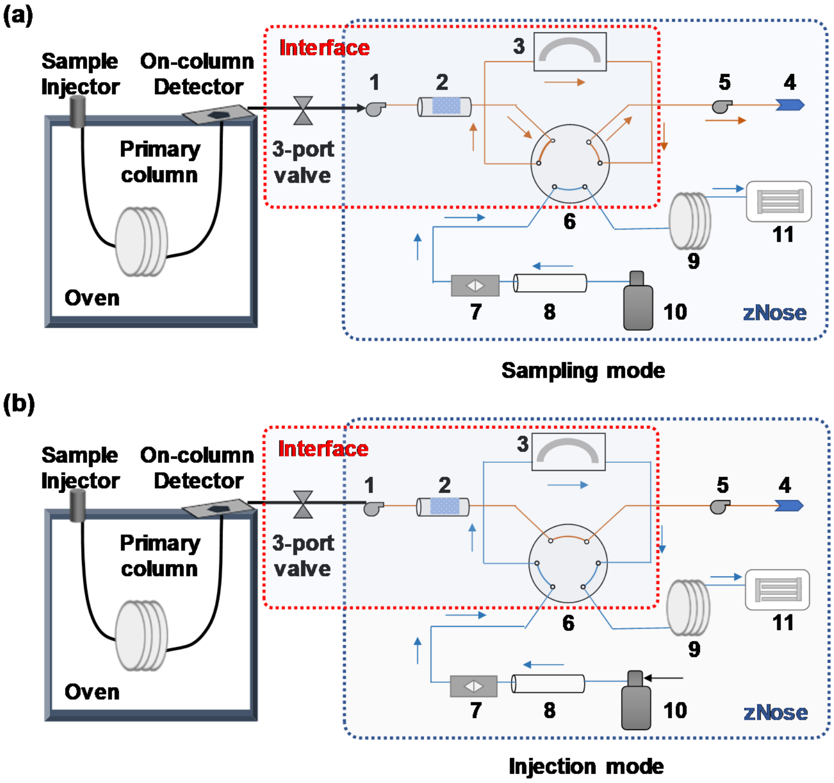

Figure S7. Cartoon showing the prototype heart-cut two-dimensional GC system. The operation procedures were composed of two steps including (**a**) sampling mode and (**b**) injection mode. The part of interface enclosed by red dotted lines consists of a 3-port valve, a sampling pump, a preconcentrator, a loop trap, and a six-port valve. (1) sampling pump; (2) preconcentrator; (3) loop trap;(4) exhaust vent; (5) vacuum pump; (6) six-port valve; (7) flow controller; (8) filter; (9) secondary column; (10) carrier gas supply; (11) SAW detector.

Initially, all the analytes were injected by a GC sample injector. Then, the operation procedures were composed of two steps.

Step 1: Sampling mode. The analytes underwent the 1D separation and passed through the on-column detector. The 3-port valve was maintained at a position of preventing flow from the 1D separation system to the 2D separation system during this period. Subsequently, the coeluted analytes, determined by the primary detector and defined as heart-cutting fraction, were pumped in by switching on the valve and trapped by the preconcentrator at room temperature. Then, sampling pump was switched off and the species to be trapped were desorbed by heating the preconcentrator to 200 °C. A sample of collector vapors was made to pass through the loop trap via a vacuum pump and reside in it for the moment. During ten seconds of sampling time, a carrier gas supplied by the helium gas cylinder flowed through a filter and a flow controller, therewith passing through the 6-port valve, 2Dseparation column and reaching the surface of SAW detector to complete the cleaning process.

Step 2: Injection mode. The heart-cutting analytes underwent the 2D separation and detection. After the sampling mode expired, the 6-port valve was switched to the position shown in Figure S7b, and the helium gas flowed through filter, flow controller and loop trap. An extremely short time was consumed to raise the temperature of loop trap to 200 °C to release the trapped vapor species into the carrier gas at this point. Whereafter, the carrier gas carried these desorbed analytes through the 6-port valve, 2Dseparation column

and SAW detector. By this means, the heart-cutting fraction from 1D separation was precisely identified.
